# Associated factors for cognition of physically independent elderly people living in residential care facilities for the aged in Sri Lanka

**DOI:** 10.1186/s12888-018-2003-5

**Published:** 2019-01-08

**Authors:** Madushika Wishvanie Kodagoda Gamage, Chandana Hewage, Kithsiri Dedduwa Pathirana

**Affiliations:** 1 Department of Nursing, Faculty of Allied Health Sciences, University of Ruhuna, Galle, Sri Lanka; 2 Department of Physiology, Faculty of Medical Sciences, University of Sri Jayewardenepura, Gangodawila, Nugegoda, Sri Lanka; 3 Department of Medicine, Faculty of Medicine, University of Ruhuna, Galle, Sri Lanka

**Keywords:** Elderly people, Cognition, Associated factors

## Abstract

**Background:**

As the elderly population and prevalence of dementia is increasing, it is necessary to have a better comprehension of the influence of specific factors on cognitive function. Dementia is not an inevitable consequence of ageing. Lifestyle factors might either increase or decrease the risk. Even though different studies have focused on individual factors, only a few studies are available which assess all these factors as a whole. Available evidence on these factors is mainly from high income countries and much less evidence is available from low and middle income countries. As cognition is critical for elderly people to engage in a physically independent life, we aimed to identify the associated factors of cognition.

**Methods:**

This was a descriptive cross sectional study performed with 421 elderly people dwelling in residential care facilities for the aged in two selected districts in the Southern Province of Sri Lanka. Cognition was assessed using the Mini Mental State Examination (MMSE). Independent sample t test, ANOVA and regression analyses were used to explore associated factors for cognition. The statistical significance was kept at bonferroni adjusted *p* < 0.004.

**Results:**

The study included elderly people with a mean age of 71.9 ± 6.7 years and of them 65.8% were females. Factors affecting higher level of cognition were, having upper secondary, advanced and higher education; being married; arriving at the facility on one’s own accord; being visited by family members; higher physical activity levels and engaging in social and leisure activities (*p* < 0.004). The factors, namely physical activity level, educational status, visits by family members and engaging in leisure activities were the predictors of cognition in the regression model.

**Conclusion:**

Though there were several factors that associated with the level of cognition such as educational status, marital status, reason for attending the facility, visits by family members, physical activity levels and participation in social and leisure activities, only the factors, such as physical activity levels, visits by family members, educational status and engaging in leisure activities were the predictors of cognition.

## Background

Population ageing, an increase in numbers of those aged 60 years and above, is a worldwide twenty-first century phenomenon [1]. This occurs due to two derivatives; increasing longevity and decrease in fertility [2]. The definition of ageing itself is arbitrary. Ageing is a biological reality which holds its own dynamic away from human control [3]. With an increase in the world’s ageing population, the number of elderly people living with dementia is also in an upward trend. This figure is projected to rise more in low and middle income countries [4].

Dementia is a progressive disorder affecting memory, other cognitive abilities and behaviour and it is the greatest global challenge in the twenty-first century [5]. Dementia is not an inevitable consequence of ageing [6]. Decline in cognitive functions with ageing is similar to other health characteristics, such as sensory and musculoskeletal functions. Cognitive functions begin to decline at a comparatively young age, and different functions decrease at different rates [3]. Cognition is a process, by which “sensory inputs are transformed, reduced, elaborated, stored, recovered and used” [7]. Cognitive impairment can be either due to normal ageing process or due to disease conditions relating to neuroanatomical, biochemical or electrophysiological changes in the brain [8]. Cognitive health promotion, that is, to maintain “brain health” with ageing has become more and more vital for the elderly [9]. But, for planning and preventive/intervention measures, identification of specific elements that may underlie cognitive decline is essential. Life style factors may modify the risk of development of dementia [6].

Though different studies have concentrated on individual factors, few studies are available which assess the factors as a whole. Positive relationship between physical activity and cognition is well addressed by many authors [10, 11]. In addition, many other factors have been assessed individually. Most of the researchers have focused their attention on the individual factors, such as age [12] and educational status [13]. A few studies have assessed the impact of marital status [14], leisure activities [15, 16], social activities [17] and sleep [18] on cognition. However, in studies, the findings were not consistent. For example, some evidence suggests that women have less age-associated cognitive decline [19] and more resilience to age-related cognitive decline than men [20], while others show more cognitive loss in females than men in the community [21] and institutions [22].

Available evidence on risk factors for dementia are mainly from high income countries and less evidence is available from low and middle income countries [6]. In Sri Lanka, population ageing is observed [2]. According to Sri Lankan norms, elderly people are looked after by their children and family members and it was longly existed [2]. However, as a result of socio demographic changes and changes in family system such as increasing number of employed women, decreasing number of children and conversion of family system from extended to nuclear families, the care for the elderly people are reducing [2]. As a result, the number of elderly people living in aged care facilities is increasing [2]. Meals, lodging, recreation, protection and other facilities for the residents are provided free of charge through different sources of funding [23]. There are caregivers in these facilities but they are not adequately trained for the purpose. Cognitive functions are particularly important for elderly people in residential care facilities as they need to have physical independence without family support.

The level of cognition and its associated factors will be different for elderly people in residential care facilities for aged compared to elderly people in the community, due to divergence in living arrangements. Previous studies have mainly focused their attention on community dwelling elderly populations [17, 20, 21]. With the growth of population ageing leading to increased demand for long term care facilities, it would be necessary to understand the combined effect of different factors rather than individual effects on their cognitive functions. It has been shown that cognitive decline is linked with physical dependency and poor quality of life [24]. Some associated factors will be modifiable factors; hence, identification of such factors will help health care professionals to use them in planning therapeutic measures to preserve cognition in the elderly.

## Methods

### Study design and setting

This was a descriptive cross sectional study. Participants were selected from the residential care facilities for aged in Galle and Matara Districts in Southern Province, Sri Lanka. Most of the residential aged care facilities are situated in Western and Southern provinces. In Sri Lanka, elderly people admitted to residential care facilities mainly due to socio economic issues [23].

### Recruitment of subjects

**Fig. 1:**
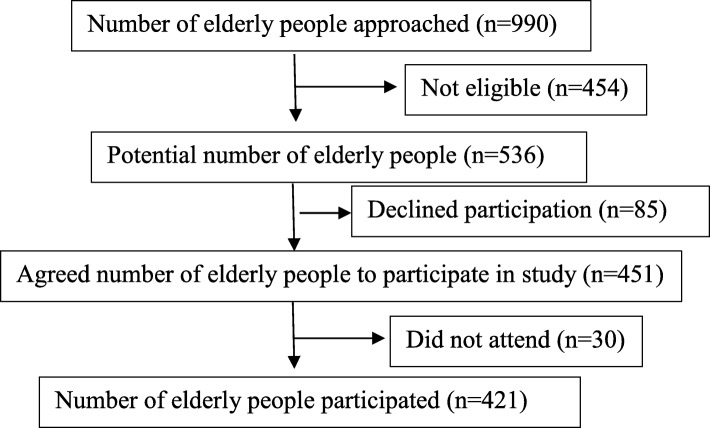
Recruitment of subjects to the study. The process of recruitment of study subjects is shown. The number of subjects approached, which was not eligible, who declined participation, who did not attend and the number of subjects who participated to the study are shown

Subjects were recruited using probability proportional to size sampling technique. First, an aged care facility was selected randomly and then, all the elderly people in that facility was screened for selection criteria. Among them, those who fulfilled the criteria and who volunteered to participate were recruited as the study sample. Method of recruitment has previously decribed in Gamage, Hewage and Pathirana [23]. The number of subjects recruited for this part of the study was 421 as shown in Fig. 1.

The sample for the analysis consisted of 421 individuals, aged 60 years and above. Participants who had psychiatric disorders, severe cognitive impairment, severe untreated vision or hearing loss and neurological and musculoskeletal disorders were excluded. Psychiatric disorders refer to any mental disorder diagnosed by a psychiatrist such as depression, bipolar affective disorder and schizophrenia. Neurological disorders refer to disorders of the nervous system diagnosed by a neurologist that affects test performance such as stroke and aphasia. Musculoskeletal disorders were the disorders in the musculoskeletal system diagnosed by a medical officer that affects test performance such as osteoarthritis, fractures and amputations. Presence of these disorders was confirmed by using health records of the participants. Those who have corrected vision worse than 6/60 were considered as having severe vision loss. Hearing loss was interviewer rated. Selected subjects were physically independent elderly people based on the score of Barthel’s index.

### Cognitive performance- mini mental state examination (MMSE)

The MMSE is a widely used and well-known questionnaire to screen cognitive impairment [25]. It is a 30 point scale and assesses five areas; orientation, registration, attention and calculation, recall and language. A score of less than 11 was considered as having severe cognitive impairment [26].

### Demographic and other factors

A pretested self-developed questionnaire was used to obtain data on demographic characteristics, presence of chronic diseases, BMI, sleep duration, presence of sleep disturbances, reason for attending facility, visits by family members and participation in leisure and social activities. One of the authors who is a registered nursing officer in Sri Lanka interviewed the subjects. Informant was used to confirm the information given by the participants.

Age, gender, educational status and marital status were asked from the participants. Age was confirmed using the participants’ identity card. Information such as presence of chronic diseases was sourced using the medical records they had. Height and weight of the participants was measured to calculate BMI. Score was categorized based on the recommended BMI cut off scores for Asian populations [27]. The number of sleeping hours participants had at night and presence of sleep disturbances was inquired from the participants.

The reason for attending the facility was considered as two factors. Whether the participants chose to admit themselves to pursue a more leisurely life away from family responsibilities and work, or whether they were compelled to be admitted due to lack of available care at home, migration of family or familial discord. Visits by family members accounted as revealed by subjects whether family members come to the facility to visit them or not.

### Social and solitary leisure activity participation

Adams, Leibbrandt and Moon mentioned, theoretically any activity can be grouped as either social or solitary activities [28]. Solitary activities included the activities that one does alone while social activities included activities done in groups. We considered the activities engaged during the past month. First, individual activities were considered and then they were allocated into types. We considered four types of solitary leisure activities [29, 30] as engagement in mental; i.e. reading books, chanting religious versus, physical; i.e. gardening and performance of exercises, productive; i.e. craft work and recreational activities; i.e. watching television, listening to radio programmes. Social activity participation included the participation in four types of activities [17] as voluntary work, social interactions, participation in community related organizations and participation in activity groups.

### Physical activity

The International Physical Activity Questionnaire modified for Elderly (IPAQ-E) was used to determine the level of physical activity. It provides continuous scores as well as categorical values. Based on their physical activity score, participants were categorized as inactive, minimally active and as having health enhancing physical activity (HEPA) level [31].

### Statistical analysis

Statistical analysis was performed using SPSS 20.0 version. Frequencies, means and standard deviations of the variables were calculated. The normality of the data was assessed. Independent sample t test and ANOVA were used. Factors that showed a significant association were used in the regression analysis. Multicollinearity was assessed using correlation analysis (r). Variance inflation factor (VIF) between variables and Durbin–Watson statistic was calculated [32]. A Bonferroni correction was applied. The number of observations was (12). Hence, the statistical significance was kept at *p* < 0.004.

## Results

Out of 421 subjects, 277 (65.8%) were women and the mean age of the participants was 71.9 ± 6.7 years. Demographic characteristics of the participants are tabulated in Table 1. Most of the participants (60.8%) had only obtained primary and lower secondary education and were unmarried (44.4%). The majority of the elderly people engaged in both solitary leisure (86.9%) and social (82.4%) activities. The majority of the participants were minimally active (63.4%) and only 16.4% had HEPA levels. The mean score of the MMSE was 22.9 ± 4.9. MMSE score ranged between 15 and 30. In our sample, 64.1% had chronic diseases; 12.8% had respiratory disorders, 32.1% had hypertension and cardiovascular disorders, 1% had gastrointestinal disorders, 4% had musculoskeletal disorders, 18.3% had diabetes mellitus, 17.8% had urinary disorders and 5.5% had other medical disorders.

**Table 1:** Demographic characteristics of the participants

Characteristic	Percentage (%)		
	All elderly people (*n* = 421)	Female (*n* = 277)	Male (*n* = 144)
Age, ≤ 70 years	180 (42.8)	129 (46.6)	51 (35.4)
Education			
Primary and lower secondary education	256 (60.8)	174 (62.8)	82 (56.9)
Upper secondary and higher Education	165 (39.2)	103 (37.2)	62 (43.1)
Marital status			
Married	131 (31.1)	80 (28.9)	51 (35.4)
Unmarried	187 (44.4)	122 (44.0)	65 (45.1)
Divorced/Separated/Widowed	103 (24.5)	75 (27.1)	28 (19.4)
BMI			
Underweight	106 (25.2)	64 (23.1)	42 (29.2)
Normal BMI	164 (39.0)	101 (36.5)	63 (43.8)
Overweight	110 (26.1)	79 (28.5)	31 (21.5)
Obesity	41 (9.7)	33 (11.9)	8 (5.6)
Reason to arrive to the facility			
By their own free will	143 (34.0)	96 (34.7)	47 (32.6)
Compelled to come	278 (66.0)	181 (65.3)	97 (67.4)
Visits by family members, Visited	240 (57.0)	159 (57.4)	81 (56.3)
Presence of chronic diseases, Present	270 (64.1)	183 (66.1)	87 (60.4)
Engagement in solitary leisure activities			
Engage	366 (86.9)	241 (87.0)	125 (86.8)
Engage in mental activities	227 (53.9)	154 (55.6)	73 (50.7)
Engage in physical activities	162 (38.5)	101 (36.5)	61 (42.4)
Engage in recreational activities	178 (42.3)	107 (38.6)	71 (49.3)
Engage in productive activities	44 (10.5)	35 (12.6)	9 (6.3)
Social activity participation			
Engage	347 (82.4)	226 (81.6)	121 (84.0)
Have social interactions	319 (75.8)	203 (73.3)	116 (80.6)
Engage in activity groups	186 (44.2)	123 (44.4)	63 (43.8)
Perform voluntary work	47 (11.2)	27 (9.7)	20 (13.9)
Participate in community organizations	28 (6.7)	26 (9.4)	2 (1.4)
Sleep duration at night, ≤ 6 h	209 (49.6)	126 (45.5)	83 (57.6)
Sleep disturbances, Present	258 (61.3)	173 (62.5)	85 (59.0)
Physical activity level			
Inactive	85 (20.2)	59 (21.3)	26 (18.1)
Minimally active	267 (63.4)	168 (60.6)	99 (68.8)
Health enhancing physical activity	69 (16.4)	50 (18.1)	19 (13.2)

### Associations of MMSE

Table 2 presents the associations between variables. The factors which had significant associations with cognition were; physical activity level, educational status, marital status, reason for attending the facility, visits by family members, engagement in solitary leisure activities and participation in social activities (*p* < 0.004).

**Table 2:** Associations between MMSE score and selected variables

Factor	All elderly	*p*	Female	*p*	Male	*p*
	Mean (SD)		Mean (SD)		Mean (SD)	
Educational status						
Primary and lower secondary education	21.3 ± 4.6	< 0.001	21.2 ± 4.6	< 0.001	21.6 ± 4.6	< 0.001
Upper secondary, Advanced level and higher education	25.4 ± 4.3		25.4 ± 4.6		25.3 ± 3.9	
Visits by family members						
Visited	24.0 ± 4.5	< 0.001	23.9 ± 4.6	< 0.001	24.4 ± 4.3	< 0.001
Did not visit	21.4 ± 5.1		21.3 ± 5.2		21.6 ± 4.8	
Reason to arrive to the facility						
By their own free will	23.9 ± 4.8	0.002	24.1 ± 4.8	0.001	23.6 ± 4.9	0.45
Compelled to come	22.4 ± 4.9		22.1 ± 5.0		22.9 ± 4.6	
Engagement in solitary leisure activities						
Engage	23.5 ± 4.8	< 0.001	23.5 ± 4.8	< 0.001	23.6 ± 4.7	0.001
Do not engage	18.8 ± 3.9		18.2 ± 4.1		19.9 ± 3.4	
Social activity participation						
Engage	23.6 ± 4.7	< 0.001	23.6 ± 4.8	< 0.001	23.8 ± 4.5	< 0.001
Do not engage	19.5 ± 4.4		19.4 ± 4.5		19.6 ± 4.3	
Marital status						
Married	24.8 ± 4.6	< 0.001	24.7 ± 4.6	< 0.001	24.8 ± 4.6	0.005
Unmarried	22.1 ± 4.9		22.1 ± 5.1		21.9 ± 4.5	
Divorced/Separated/Widowed	22.1 ± 4.7		21.8 ± 4.8		22.9 ± 4.4	
Physical activity level						
Inactive	17.0 ± 2.5	< 0.001	17.1 ± 2.6	< 0.001	16.8 ± 2.3	< 0.001
Minimally active	23.6 ± 4.2		23.3 ± 4.4		24.1 ± 3.9	
Health enhancing physical activity	27.5 ± 2.3		27.8 ± 1.8		26.7 ± 3.1	

*SD- Standard deviation

The level of cognition was not associated with other factors, such as age, gender, BMI, presence of diseases, presence of sleep disturbances and sleep duration at night (*p* > 0.004). Though marital status and reason for arrive at the facility were associated factors for cognition among women, this was not observed among men. Those who were married had a significantly higher MMSE score than unmarried and divorced/widowed/separated participants. Those who had HEPA level had a significantly higher MMSE score than inactive and minimally active elderly people. When leisure and social activity types were considered, those who engaged in physical and mental solitary leisure activities had a significantly higher MMSE score than their counterparts (*p* < 0.001) but we did not observe this significant difference between those who engaged in recreational and productive solitary activities and those who did not (*p* > 0.004). Further, those who had social interactions, participated in activity groups, voluntary work and community related organizations had a significantly higher MMSE score than their counterparts (*p* < 0.001).

We included the associated variables, namely educational status, marital status, reason for attending the facility, visits by family members, engagement in solitary leisure activities, participation in social activities and physical activity level, in the regression analysis. Correlations between variables are shown in Table 3. Predictors of cognition in regression model are tabulated in Table 4. The same variables were entered to regression analysis for women but in men marital status and reason for attending the facility were excluded as they were not associated variables.

**Table 3:** Correlation between variables

	MMSE	Educational status	Reason to arrival to facility	Social activity participation	Engagement in leisure activities	Visits by family members	Physical activity level	Marital status
MMSE	1.000							
Educational status	.399	1.000						
Reason to arrival to facility	−.150	−.071	1.000					
Social activity participation	−.325	−.051	.186	1.000				
Engagement in leisure activities	−.323	−.109	.144	.321	1.000			
Visits by family members	−.266	−.108	.086	.204	.176	1.000		
Physical activity level	.654	.325	−.128	−.291	−.232	−.128	1.000	
Marital status	−.213	−.144	.051	.151	.073	.052	−.249	1.000

**Table 4:** Associated variables of cognition

		Determinant	Standardized Beta coefficient	*p*	Adjusted R^2^	F
All elderly people	MMSE score	Physical activity level	0.54	< 0.001**	0.513	111.45
		Educational status	0.19	< 0.001**		
		Visits by family members	−0.15	< 0.001**		
		Engaging in leisure time activities	−0.15	< 0.001**		
		Constant		< 0.001**		
Female	MMSE score	Physical activity level	0.55	< 0.001**	0.543	83.09
		Educational status	0.20	< 0.001**		
		Visits by family members	−0.17	< 0.001**		
		Engaging in leisure time activities	−0.17	< 0.001**		
		Constant		< 0.001**		
Male	MMSE score	Physical activity level	0.55	< 0.001**	0.412	51.10
		Educational status	0.21	0.002**		
		Constant		< 0.001**		

*Significance value p < 0.004; **

About 51.3% of variance in cognition was explained by the observed following variables; physical activity level, visits by family members, educational status and engaging in solitary leisure activities. In male participants, 41.2% of variance in cognition was explained by the variables such as physical activity level and educational status while in women 54.3% of variance in cognition was explained by the variables such as physical activity level, educational status, visits by family members and engaging in leisure activities.

## Discussion

With population ageing, the number of people living with dementia is in an upward trend and it is expected to continue to escalate in low and middle income countries. As dementia is not an inevitable consequence of ageing, life style factors might modify the risk of dementia. It was calculated that more than a third of cases of dementia might be preventable theoretically by eliminating most potent factors for dementia. However, available evidence has focused on high income countries and less evidence is available from low and middle income countries [6]. In this study, we aimed to identify the associated factors of cognition of elderly people living in residential care facilities for aged, as the demand for long term care of elderly is increasing [33].

In our study, the majority were female, had obtained only primary and lower secondary education and were unmarried. The sample has more females due to the fact that females live longer than men [34]. In Sri Lanka, the life expectancy of females is longer (75.8 for females and 71.2 for males in 2000) than men [2]. Most of the participants had engaged in solitary leisure and social activities and were minimally active. There were several factors that associated with the level of cognition such as physical activity level, educational status, marital status, reason for attending the facility, visits by family members and engagement in social and leisure activities. Among them only the factors such as physical activity level, visits by family members, educational status and engaging in leisure activities were the predictors of cognition in regression model.

Variables affecting the cognition have been different in other studies [21, 35–38]. Among different sociodemographic factors only educational achievements significantly associated with cognitive disorder while other factors such as age, gender, employment history, ethnicity, marital status did not in a Malaysian study [35]. In a South African study, those who were at younger age, who were married, who gained secondary level education or higher, who had moderate or high physical activity level had higher cognitive functioning [36]. In an Indian study, cognitive impairment was more likely to be among those who were old-old, who were illiterate and were widowed [37]. In a Brazilian study, those who had systemic arterial hypertension and those who had been institutionalized on their own will were found to be less likely to be cognitively impaired [38]. In Maroof et al., [21] study, age, gender, marital status and literacy level significantly associated with having cognitive impairment.

Age is a well-documented factor that affects cognition [12, 36, 38] and it is the greatest non modifiable risk factor [6]. In two studies, age did not exert a significant influence on cognition [35, 39]. With bonferroni adjusted *p* value (*p* < 0.004) in our study, we did not observe a significant difference in the cognition based on age but the association had a p value of 0.018. Changes in the structure and function of the brain might correlate with these age-related cognitive changes [12]. Livingston et al., [6] suggested that age as a less powerful risk factor when it was taken into consideration with other comorbidities. Torres et al., [40] has shown that less education in early life depicts a much stronger influence than age for age related cognitive decline. Further, this is in line with previous findings in ageing Brazilian populations [41]. Hence, as our sample constituted of more elderly people with less education and as we have considered age with other comorbidities, we might have lost the significant impact of age on cognition.

We did not observe a statistically significant difference in cognition between males and females. Few studies mentioned significant difference in cognition based on gender [21, 22, 39] while many reported no significant difference [20, 35, 38]. Sex differences in cognitive ageing may be due to differences in brain structure and function [21].

In our study, educational status was a predictor of cognition in regression model for both males and females. Those who had obtained upper secondary, advanced level and higher education had significantly higher level of cognition than the other group. Similar findings were observed in three other studies saying that cognitive status significantly differed based on schooling [42–44]. A Malaysian study showed education attainment as the only sociodemographic factor that showed significant association with cognitive disorder [35]. Three possible mechanisms have explained for having lower rates of cognitive decline in elderly people with higher level of education. The first mechanism is that people with lower education have a higher risk of damage to central nervous system; second, people with higher education may have more neuronal reserve capacity; and third, people with higher education may have the ability to generate better strategies of compensation [45].

A Chinese study showed a higher chance of cognitive deterioration in elderly males who were single and widowed, compared to married, but this was not evidenced among women [14]. However, we observed this finding in opposite direction. We observed a significant difference in women but not in men and this might be due to considering bonferroni adjusted *p* value (*p* < 0.004) or females gaining more emotional stress by loss of their partner compared to males. Being married positively associated with cognition [36], while being widowed showed a higher probability of cognitive impairment [22, 37]. A higher prevalence of cognitive impairment was observed among elderly people who live without a partner in institutions [38]. Married elderly gain better mental conditions through sharing their life with a partner. Emotional stress in divorced or widowed people due to unexpected life events might negatively affect their cognition [35].

Data available on relationship between BMI and cognition is not consistent. Some studies suggest obese individuals are at a lower risk of cognitive impairment [46, 47] while others show overweight and obese individuals are known to be at a higher risk for dementia [48]. In our study, cognition did not significantly vary based on BMI similar to what Zhou et al., [49] had found. The association of cognition with BMI is bidirectional. It might be that either high or low BMI leading to cognitive impairment or vice versa.

In our study, those who chose to be admitted to facility as their wish had a higher level of cognition. It might be due to less psychological stress. Previous studies have shown psychological stress [50] was associated with increased risk of dementia. From our study, we cannot determine the cause and effect of whether voluntary admission improves cognition or those who had cognitive impairment were compelled to come to the care facilities. Past literature supports on both aspects. de Andrade et al., [38] depicted institutionalization by own choice as a protective factor for cognitive impairment. Pereira and Besse [51] found a higher level of functional independency among elderly who were self-institutionalized which will subsequently lead to better cognition. In contrast, a systemic review mentioned that those who had cognitive impairment had a higher chance of getting institutionalized and cognitive impairment was a predictor of institutionalization [25].

Elderly living in elderly homes experience loneliness. However, this depends on number of family visits. Further, elderly people in care facilities prefer if they are visited by their family members as frequently as possible [52]. In our study, those who were visited by family members had a higher level of cognition. It was a predictor of cognition among women in regression model. This might be due to lessening of the feeling of being isolated. Either visits by family members resulted in higher cognition or those who were cognitively impaired were not visited by family members cannot be determined by our study due to the nature of the study design. Social isolation is one of the risk factors for development of dementia [6], and loneliness [53] was associated with increased risk of dementia. Greater purpose in life [54] was associated with reduced risk of dementia. Nevertheless, individuals who were mentally and physically declined, who had impairments in cognition, were less likely to be visited by their family members [52].

Previous research has focused attention on different diseases which lead to cognitive decline [6, 38]. The association of diabetes mellitus and mid-life hypertension with cognitive impairment has been previously observed [55, 56]. We did not observe a significant difference in the levels of cognition based on the types of diseases, such as diabetes mellitus, cardiovascular disorders and other disorders. It was hypothesized that anomalies in peripheral insulin cause decreased production of brain insulin which results in impairment of amyloid clearance which leads to impairment in cognition [57]. A review showed that diabetes increased the risk of conversion of mild cognitive impairment to dementia [58].

In recent decades, the attention on the protective role of leisure activities against the occurrence of dementia is increasing [59]. Paillard-Borg et al., [29] examined five types of leisure activities namely mental, social, physical, productive and recreational. We used this as the classification but we considered social activities as a separate entity. We found those who participated in solitary leisure activities had significantly higher level of cognition than those who do not participate. It was a predictor of cognition among women in regression model. When elaborated, those who participate in physical and mental solitary leisure activities had a significantly higher level of cognition than those who did not. According to Paillard-Borg et al., [29], there is an association between cognitive functioning and participation in mental, social and productive activities.

Physical leisure activities promotes physical engagement while mental leisure activities do mental stimulation which in turn improves cognition. Intellectual stimulation has been considered as a factor that enhance cognitive reserves [6]. The reason for not observing a significant difference in cognition in those who engaged in productive activities might be due to having a low proportion of participants in that category. As to Paillard-Borg et al., [29] productive activities are cognitively stimulating but recreational activities are less so [29]. This could be the reason for not observing a significant difference in cognition based on participation in recreational activities in our study.

We cannot determine whether poor cognition led to poor participation in leisure activities or vice versa. It was mentioned that participants with dementia were less active in mental, social and productive activities. They speculated it as either experiencing loneliness or living with dementia might prevent elderly people from being active in leisure activities [29]. In contrast, plenty of studies show the protective role of leisure activities on cognition [59–61]. The mechanism underlying the protective impact of leisure activities on cognition is still uncertain, a number of theories have been proposed. Krammer et al., [62] suggested that leisure activities make neurological processes efficient, adaptive and plastic which in turn would be helpful in coping with progressing cognitive decline in dementia. However, larger samples and long follow-up are required in order to confirm the protective role of leisure activities [59].

Social engagement reflects partaking different social activities and having social contacts [63]. Fu, Li and Mao [17] have categorized social activities into five groups i.e.; participating in community related organizations, voluntary work, hobby groups and sports groups and having connections with friends. These are the areas which we used for categorization. In our study, we merged participation in hobby groups and sports groups to one variable and it was named as participation in activity groups. Elderly people who participated in social activities had better cognition than those who did not. Elderly people who participated in voluntary work, had social interactions, participated in activity groups and community organizations had significantly higher level of cognition than those who did not.

It is uncertain whether cognitive decline is the cause of reduced socialization or vice versa [6]. On one direction, social activities reduce social isolation and thereby improve cognition [28] and it is potentially a modifiable risk factor [64]. On the other hand, it may be that subjects with cognitive impairment have poor participation in social activities but little evidence is available for reverse causation [65]. Recent evidence proposes that older people who are more socially engaged have a higher level of cognitive function [17, 64, 66]. Fu, Li and Mao have stated several possible explanations for the relationship between social activities and cognitive function [17]. Social activities may help to improve cognition by enabling more complex and compound thinking [67], maintaining or expanding the social network [66, 68] and providing a greater sense of purpose [69].

We did not observe a significant difference in cognition based on sleep duration and with bonferroni adjusted *p* value, presence of sleep disturbances was not an associated factor. The relationship between sleep and cognition is bidirectional. Cognitive decline may lead to poor sleep or vice versa. Sleep disturbances significantly increase cognitive decline [70, 71]. It has been hypothesized that sleep deprivation aggravate the neuropathological processes leading to amyloid depositions [72, 73]. It has observed that alterations in sleep duration occur prior to the appearance of cognitive symptoms in Alzheimer’s disease [74].

Physical activity is a cognitive reserve enhancing factor and physical inactivity is a cardiovascular risk factor for dementia [6]. We observed a significant difference in MMSE score among three physical activity groups which are inactive, minimally active and HEPA. Physical activity was a predictor of cognition in the regression model. The relationship between physical activity and cognition is bidirectional. One can argue, higher physical activity leads to higher cognition. Other might argue that higher cognition makes an individual more physically active. We cannot determine the cause and effect due to the cross sectional nature of the study, but it is more plausible to say that high physical activity levels lead to improvements in cognition [75–79]. Changes in hormone levels, improvement in cerebral blood flow and an increase in number of neuronal synapses, brain volume and plasticity are thought to be contributory in physiological benefits of physical activity [10]. A review of Lista and Sorrentino discussed on supramolecular and molecular mechanisms of how physical activity prevents cognitive decline. These two biological mechanisms included neurogenesis or neural cell proliferation, synaptogenesis, angiogenesis and the role of brain-derived neurotrophic factor (BDNF) [80].

The cognitive abilities which persist steady, get worse or get advance with age will depend on the health of the brain and body. Further, skills that we are practicing in our day to day lives will also contribute for it [81]. Livingston et al., [6] suggested that 35% of dementia is due to a combination of nine risk factors. They had mentioned having midlife hypertension and obesity, having hearing loss, having depression in late life, smoking, having diabetes, being physically inactive, being educated to a maximum of 11–12 years and being socially isolated as risk factors. In our study, physical activity level, educational status, visits by family members and engaging in leisure time activities were the predictors in regression model with 51.3% of the variance. Other factors that have not been followed or are unexplained in the study may have contributed to the remainder of the variance in the cognition, for which further investigations are necessary. Some of the factors that Livingston et al., [6] mentioned were not investigated in our study but in both physical activity and educational status are mentioned. The associated factors for cognition in western countries might be different from Asian countries. Further, associated factors for cognition in elderly people in aged care facilities will be different from community dwelling elderly people as their living arrangements are different. As a result of population ageing, demand for long term care has increased [33], and hence it might be worthwhile to investigate these factors to establish prevention strategies.

Already, cognitive stimulating lifestyles have been identified as reducing cognitive deterioration. Among them, cognitive training, physical activity, social engagement and nutrition play a vital role [82]. Currently, available literature suggests the importance of magnesium [83] to reduce cognitive deterioration. A meta-analysis suggested that vitamin D deficiency increases the risk of cognitive impairment [84]. Dickens et al., [85] mentioned, though cross sectional studies had proved the association between vitamin D and cognitive decline, no prospective studies are available and they recommend further studies. Durga et al., [86] had shown that folic acid supplementation for 3 years, significantly improved cognitive function. Physical activities such as Tie chi exercises have shown positive effects on cognitive functions [87]. Hanna-Pladdy and MacKay suggested a strong predictive effect of high musical activity throughout the life on preservation of cognitive functioning in advanced age [88]. Working on cognitive stimulating activities such as crossword puzzles has been associated with preservation of cognition in older adults [89]. Further, depression has been proposed to be a risk factor for dementia [90]. In our study, we excluded elderly people who had diagnosed as having depression. However, treating depression might be useful to improve cognition of elderly people via treatment with antidepressants [90]. If a person can delay the onset of dementia, that leads to have a more fulfilling life for a longer duration with less time suffering from dementia [91]. In other studies as well as in our study, we found participation in physical activities, social and leisure activities as associated factors of cognitive decline. Hence, it may be beneficial to address these in elderly people living in care facilities and encourage them in participation in physical, social and leisure activities.

### Strengths and limitations

This study provides evidence on associated factors for cognition of elderly people living in residential care facilities for aged in Sri Lanka, which is an Asian country as well as a lower middle income country. We had several limitations. One is our study was cross sectional in nature so we cannot determine the cause and effect. There were some reviews on some aspects to identify direction of the relationship but on some aspects it was not for example; visits by family members. Minimal MMSE score in our sample was 15 and this might be due to inclusion of only physically independent elderly people. Memory problems and recall bias might have affected on reliability of the answers. We recommend interventional or case control studies to confirm these findings.

## Conclusion

Though there were several factors that associated with the level of cognition such as physical activity level, educational status, marital status, reason for attending the facility, visits by family members, participation in social activities, engagement in solitary leisure activities, only the factors such as physical activity, visits by family members, educational status, engaging in solitary leisure activities were the predictors of cognition. These factors need to be addressed in caring for elderly people in care facilities.

## Abbreviations

ANOVA: Analysis of Variance
BDNF: Brain-Derived neurotrophic factor
HEPA: Health enhancing physical activity
IPAQ-E: International physical activity questionnaire-elderly
MMSE: Mini Mental State Examination
SD: Standard Deviation
SPSS: Statistical Package for Social Sciences
VIF: Variance inflation factor
